# mRNA-Expression of *KRT5* and *KRT20* Defines Distinct Prognostic Subgroups of Muscle-Invasive Urothelial Bladder Cancer Correlating with Histological Variants

**DOI:** 10.3390/ijms19113396

**Published:** 2018-10-30

**Authors:** Markus Eckstein, Ralph Markus Wirtz, Matthias Gross-Weege, Johannes Breyer, Wolfgang Otto, Robert Stoehr, Danijel Sikic, Bastian Keck, Sebastian Eidt, Maximilian Burger, Christian Bolenz, Katja Nitschke, Stefan Porubsky, Arndt Hartmann, Philipp Erben

**Affiliations:** 1 Institute of Pathology, University of Erlangen-Nuremberg, 91054 Erlangen, Germany; robert.stoehr@uk-erlangen.de (R.S.); arndt.hartmann@uk-erlangen.de (A.H.); 2 STRATIFYER Molecular Pathology GmbH, 50935 Cologne, Germany; ralph.wirtz@stratifyer.de; 3 Institute of Pathology at the St Elisabeth Hospital Köln-Hohenlind, 50935 Cologne, Germany; Sebastian.eidt@stratifyer.de; 4 Department of Urology, University Medical Centre Mannheim, Medical Faculty Mannheim, University of Heidelberg, 68167 Mannheim, Germany; matthias.gross-weege@umm.de (M.G.-W.); katja.nitschke@medma.uni-heidelberg.de (K.N.); Philipp.Erben@medma.uni-heidelberg.de (P.E.); 5 Department of Urology, University of Regensburg, 93053 Regensburg, Germany; Johannes.breyer@ukr.de (J.B.); wolfgang.otto@ukr.de (W.O.); maximilian.burger@ukr.de (M.B.); 6 Department of Urology and Pediatric Urology, University Hospital Erlangen, 91058 Erlangen, Germany; danijel.sikic@uk-erlangen.de (D.S.); Bastian.keck@web.de (B.K.); 7 Department of Urology, University of Ulm, 89081 Ulm, Germany; Christian.Bolenz@uniklinik-ulm.de; 8 Department of Pathology, University Medical Centre Mannheim, Medical Faculty Mannheim, University of Heidelberg, 68167 Mannheim, Germany; Stefan.porubsky@medma.uni-heidelberg.de

**Keywords:** Bladder cancer, muscle-invasive bladder cancer, molecular diagnostics, molecular subtyping, *KRT5*, *KRT20*

## Abstract

Recently, muscle-invasive bladder cancer (MIBC) has been subclassified by gene expression profiling, with a substantial impact on therapy response and patient outcome. We tested whether these complex molecular subtypes of MIBC can be determined by mRNA detection of keratin 5 (*KRT5*) and keratin 20 (*KRT20*). Reverse transcriptase quantitative polymerase chain reaction (RT-qPCR) was applied to quantify gene expression of *KRT5* and *KRT20* using TaqMan^®^-based assays in 122 curatively treated MIBC patients (median age 68.0 years). Furthermore, in silico analysis of the MD Anderson Cancer Center (MDACC) cohort (GSE48277 + GSE47993) was performed. High expression of *KRT5* and low expression of *KRT20* were associated with significantly improved recurrence-free survival (RFS) and disease-specific survival disease specific survival (DSS: 5-year DSS for *KRT5* high: 58%; 5-year DSS for *KRT20* high: 29%). *KRT5* and *KRT20* were associated with rates of lymphovascular invasion and lymphonodal metastasis. The combination of *KRT5* and *KRT20* allowed identification of patients with a very poor prognosis (*KRT20*^+^/*KRT5*^−^, 5-year DSS 0%, *p* < 0.0001). In silico analysis of the independent MDACC cohorts revealed congruent results (5-year DSS for *KRT20* low vs. high: 84% vs. 40%, *p* = 0.042). High *KRT20*-expressing tumors as well as *KRT20*^+^/*KRT*^−^ tumors were significantly enriched with aggressive urothelial carcinoma variants (micropapillary, plasmacytoid, nested).

## 1. Introduction

Urothelial bladder cancer (UBC) is one of the 10 most common malignancies worldwide, with nearly 386,000 new cases and nearly 150,200 deaths per year [1]. Non-muscle-invasive bladder cancer (NMIBC) variants (70%) are not immediately life-threatening but often progress, while muscle-invasive bladder cancer (MIBC) tumors account only for nearly 30%, but are responsible for most deaths [2]. Due to the high cost of treatment modalities and the often necessary lifelong surveillance, UBC is one of the most expensive tumor entities [3].

The current standard of care in MIBC is radical cystectomy with perioperative platinum-based chemotherapy in selected cases. At present, clinical management of MIBC suffers from two major problems: First, the therapy selection is heavily influenced by a limited clinicopathological staging system, resulting in high rates of inadequate treatment [4]. Second, due to limited insight into molecular variants, it is not yet possible to identify potential (chemo)therapy responders [2,5]. Therefore, several groups have started to characterize UBC by gene expression profiling, as was previously done for breast cancer [6,7], which identified highly prognostic molecular signatures [5,8,9,10,11,12,13,14,15,16,17,18,19]. The MD Anderson Cancer Center (MDACC) subtypes resembled those identified for breast cancer and showed typical mRNA expression profiles of basal and luminal markers, with keratin 5 (*KRT5*) expression being highly upregulated in basal and keratin 20 (*KRT20*) being upregulated in luminal tumors. The third subtype (p53-like) is characterized by either a luminal or basal expression profile and an activated p53 wild-type pathway, which might be caused by prominent immune cell infiltration [14,20]. Recently, we demonstrated the high prognostic relevance of assessing *KRT5* and *KRT20* expression in high-risk NMIBC [21]. High *KRT5* mRNA expression identified a subgroup of nonluminal NMIBC that showed superior recurrence-free survival (RFS) and progression-free survival (PFS) despite being World Health Organization (WHO) grade 3 and stage pT1. In contrast, NMIBC with high *KRT20* mRNA expression was accompanied by significantly increased rates of tumor progression.

Here, we tested whether molecular subtyping by *KRT20* and *KRT5* mRNA expression is also applicable in MIBC. The main aim of this study was to prove the possibility and feasibility of introducing those two markers into the clinicopathological routine.

## 2. Material and Methods

### 2.1. Patient Population, Specimen Collection, and Histopathological Evaluation

Formalin-fixed paraffin-embedded (FFPE) tumor tissue samples were obtained from 169 patients with histologically confirmed MIBC (pT2-4) who were treated with radical cystectomy in conjunction with bilateral lymphadenectomy at a single center between 1999 and 2007 by 2 oncological surgeons with substantial cystectomy experience. Thirty-two patients received adjuvant platinum-containing chemotherapy (in the final study cohort, 22 patients received adjuvant platinum-containing chemotherapy). None of the patients underwent neoadjuvant radiation or chemotherapy. Hematoxylin and Eosin stained HE sections were reevaluated according to the 2017 Union internationale contre le cancer (UICC) staging manual and graded according to the common grading systems (World Health Organization1973 and 2016) by 3 experienced uropathologists (A.H., S.P., M.E.) [22]. Primary squamous cell carcinomas, pure neuroendocrine carcinomas, tumors originating from other organs (metastases or arising from neighboring organs), samples with low calmodulin 2 (CALM2) housekeeping gene expression (Ct values ≥ 28.0) and cases with missing follow-up data were excluded (*n* = 47). The final cohort consisted of 122 patients. The median follow-up period was 26.5 months (range 0.7–180.8 months). Follow-up data were achieved from local tumor registries and clinical case files, and by telephone calls to last known treating private practices.

In total, 59 patients had a recurrence: 44 patients with distant metastases, 5 patients with isolated local recurrences with delayed distant metastases, and 10 patients with co-occurrence of local and distant recurrence. The date of recurrence was defined as first confirmation of local and/or distant metastasis. Most recurrences were confirmed by computed tomography, but due to the retrospective nature of this study, in several cases we did not know the modality of recurrence detection. None of the patients received local resection of local recurrences or distant metastases.

All patients gave informed consent, and the study was approved by the institutional review board under numbers 2013-517N-MA (approval date: 21.02.2013) and 2016-814R-MA (approval date: 05.04.2016). To validate RT-qPCR-data, array gene expression data (Illumina HumanHT-12 WG-DASL V4.0 R2 expression beadchip) of 44 MIBC patients from the MDACC cohort (GSE48276) were analyzed (median age 67.6, range 41–89.6 years) [14]. 

### 2.2. RNA Isolation from FFPE Tissue

RNA was extracted from FFPE tissue using 10 μm sections, which were processed in a fully automated manner by a commercially available bead-based extraction method (XTRACT kit; STRATIFYER Molecular Pathology GmbH, Cologne, Germany). RNA was eluted with 100 μL elution buffer and RNA eluates were analyzed. The section was taken from a paraffin block containing a tumor area of at least 5 × 5 mm with a total tumor content of at least 30% tumor cells.

### 2.3. mRNA Quantification by RT-qPCR

RT-qPCR was applied for relative quantification of *KRT5* and *KRT20* mRNA as well as *CALM2* (calmodulin 2; housekeeping gene) expression by using gene-specific TaqMan^®^-based assays as described previously [21,23]. *CALM2* is a stably expressed gene among breast cancer tumor tissue samples and has been applied successfully to bladder cancer specimens [21,24,25]. Each patient sample or control was analyzed in triplicate. Experiments were run on a Siemens Versant (Siemens, Germany) according to the following protocol: 5 min at 50 °C, 20 s at 95 °C, followed by 40 cycles of 15 s at 95 °C and 60 s at 60 °C. Forty amplification cycles were applied and the cycle quantification threshold (Ct) values of 3 markers and 1 reference gene for each sample were estimated as the mean of the 3 measurements. Ct values were normalized by subtracting the Ct value of the housekeeping gene CALM2 from the Ct value of the target gene (ΔCt).

### 2.4. Statistical Analysis

All *p*-values were calculated 2-sided, and values of <0.05 were considered to be significant. Survival analyses were performed by univariate Kaplan–Meier regressions and tested for significance with the log-rank. Results were considered to be significant if the test revealed significance levels <0.05. Multivariate analyses were performed by Cox proportional hazard regression model, including all relevant clinicopathological characteristics (pT-Stage, pN-Stage, lymphovascular invasion (L), blood vessel invasion (V), age, gender, receipt of adjuvant platinum-containing chemotherapy, status of resection margins, and tumor grading (WHO 2016 and WHO 1973)). Statistical analyses of numeric continuous variables were performed by nonparametric tests (Wilcoxon rank-sum test, Kruskal–Wallis test). Contingency analysis of nominal variables was performed by Pearson’s chi-squared test. Correlation analysis of numeric continuous variables was performed using Spearman rank correlations. All statistical analyses were performed with GraphPad Prism 7.2 (GraphPad Software Inc., La Jolla, CA, USA) and JMP SAS 13.2 (SAS, Cary, NC, USA).

## 3. Results

### 3.1. Clinicopathological Data and Expression of KRT5 and KRT20 mRNA in MIBC

The distribution of clinicopathological data of the entire cohort and respective *KRT*-expression subgroups (*KRT5* high vs. low; *KRT20* high vs. low; Epi-Typer Class 1, Epi-Typer Class 2) including age, gender, pT-Stage, pN-Stage, and grading (WHO 1973, WHO 2004/2016) is depicted in Table 1.

**Figure 1:**
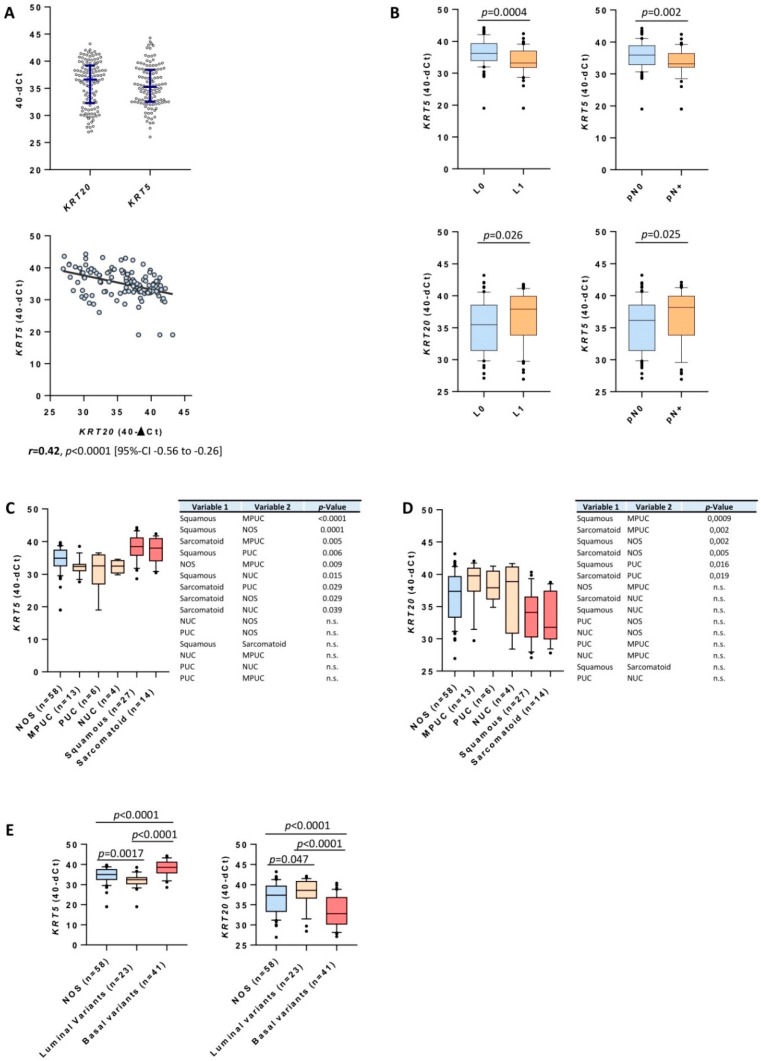
(**A**) Data distribution of *KRT5* and *KRT20* mRNA levels in patients with muscle-invasive bladder cancer (MIBC) treated with radical cystectomy and correlation of *KRT5* and *KRT20* mRNA levels. Blue bars within the boxplot indicate median value and 25%/75% quartiles. The black line in the correlation plot indicates the strength of correlation. (**B**) Correlation of *KRT5* mRNA expression levels with N-stage and lymphovascular invasion (L; L1 = lymphovascular invasion present; L0 = lymphovascular invasion absent; pN0 = no lymphnode metastasis present; pN+ = lymphnode metastasis present). High expression of *KRT5* mRNA is associated with significantly lower rates of LVI and nodal metastasis. (**C**,**D**) Distribution of *KRT5* and *KRT20* in conventional (not otherwise specified; NOS), micropapillary (MPUC), plasmacytoid (PUC), nested (NUC), squamous, and sarcomatoid differentiated urothelial carcinomas. (**E**) Distribution of *KRT20* and *KRT5* stratified by conventional urothelial carcinomas, luminal variants (including nested, plasmacytoid, and micropapillary carcinomas), and basal variants (including squamous and sarcomatoid carcinomas).

Data distribution of normalized *KRT5* and *KRT20* expression levels had a broad dynamic range (Figure 1A). Expression of *KRT5* and *KRT20* correlated inversely (*r* = −0.42, *p* < 0.0001; Figure 1A). Consistent with previous studies, there was a significant association between high *KRT5* expression and squamous and sarcomatoid differentiation (Figure 1C) [14]. Tumors with variant histology (micropapillary, nested, plasmacytoid) showed an interesting keratin expression pattern: they showed high expression of *KRT20*, while the expression of *KRT5* was very low in these cases (Figure 1C,D). High expression of *KRT5* was associated with a lower rate of lymphovascular invasion (LVI) (*p* = 0.0004) and lymphonodal metastasis (*p* = 0.002; Figure 1B), while high *KRT20* expression correlated positively with LVI/nodal status (Figure 1B). Furthermore, luminal bladder cancer variants (micropapillary, plasmacytoid, nested) exhibited significantly lower levels of *KRT5* and significantly higher levels of *KRT20* expression than conventional UBC or basal variants (sarcomatoid, squamous; Figure 1E).

### 3.2. KRT5 and KRT20 mRNA Expression Defines Highly Prognostic Relevant Subgroups of MIBC

**Figure 2:**
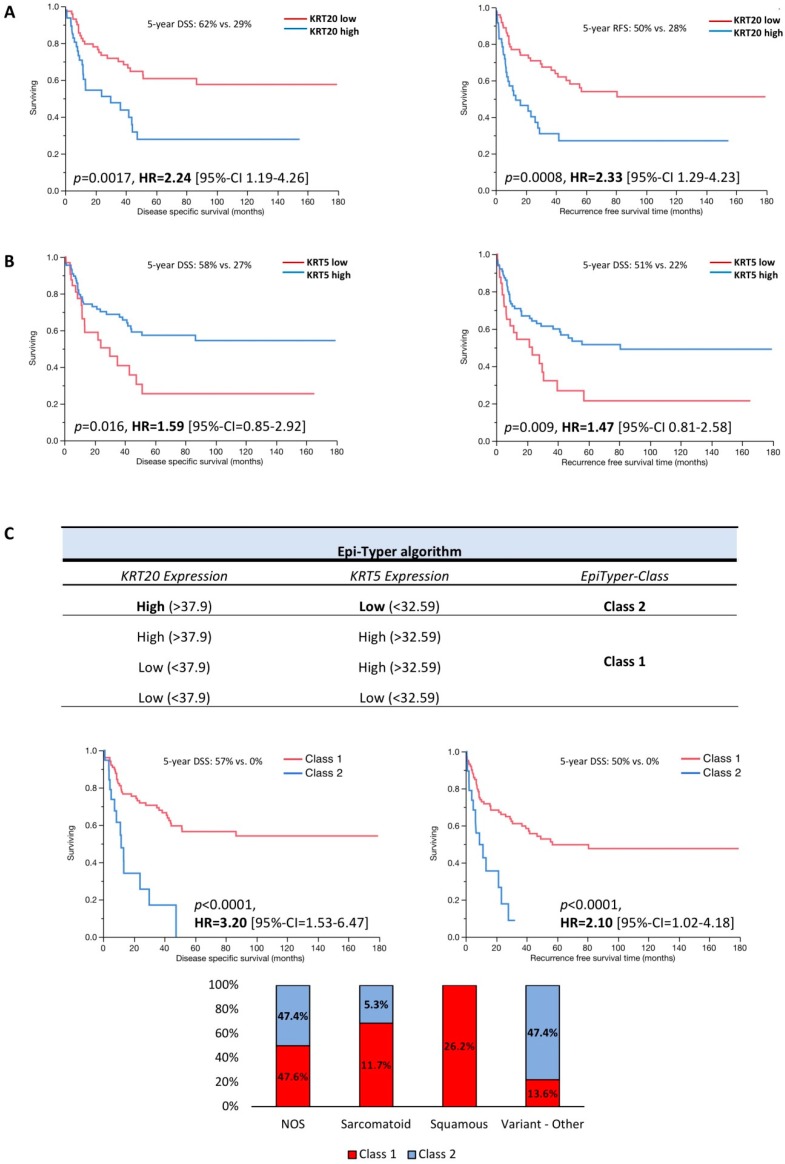
Kaplan–Meier analysis for recurrence-free survival (RFS) and disease-specific survival (DSS) based on (**A**) *KRT20* and (**B**) *KRT5* mRNA expression levels. (**C**) Epi-Typer algorithm and Kaplan–Meier analysis in the MIBC cohort for RFS and DSS based on marker combination (Epi-Typer) of *KRT5* and *KRT20* mRNA expression levels (red color: tumors within Epi-Typer Class 1; blue color: tumors within Epi-Typer Class 2).

As shown in Figure 2A,B the differential expression of *KRT5* and *KRT20* clearly defines two distinct subgroups. High *KRT20* mRNA expression was significantly associated with worse RFS (multivariate hazard ratio (HR) = 2.33) and DSS (multivariate = HR 2.24; Figure 2A, Supplementary Table S1). Low *KRT5* expression level was associated with unfavorable RFS (multivariate HR = 1.47) and DSS (multivariate HR = 1.59; Figure 2B, Supplementary Table S1). Next, an algorithm (Epi-Typer) based on the above calculated cutoffs for *KRT5* and *KRT20* mRNA expression was used to further subclassify the tumors, as depicted in Figure 2C. *KRT5* and *KRT20* cutoffs were calculated by a predictive monoforest algorithm stratified by disease-specific survival status (disease-specific death vs. no disease-specific death). There was no statistically significant difference between the *KRT5*^+^/*KRT20*^−^ and *KRT5*^+^/*KRT20*^+^ subtypes with regard to RFS and DSS when these two groups were summarized to the *KRT5*^+^/*KRT20*^+^/^−^ phenotype (data not shown). The *KRT20*^+^/*KRT5*^−^ (class 2) subgroup showed a very poor prognosis with 5-year RFS (multivariate HR = 2.10; Figure 2C) and DSS of 0% (multivariate HR = 3.20; Figure 2C), whereas the *KRT5*^+^/*KRT20*^+^/^−^ (class 1) subgroup showed a favorable prognosis with 5-year RFS of 52% and 5-year DSS of 58% (Figure 2C).

### 3.3. Multivariate Data Analysis

Multivariate Cox–proportional hazard models were calculated including fixed clinicopathological variables: pT-Stage, pN-Stage, Grading WHO 1973, Grading WHO 2016 (no impact; all included tumors were high grade), lymphovascular invasion, blood vessel invasion, age at cystectomy, gender, resection margin status, receipt of adjuvant platinum-containing chemotherapy, and presence of urothelial carcinoma in situ. Models were calculated for each respective cutoff group (*KRT5* high vs. low, *KRT20* high vs. low, Epi-Typer classes). Detailed multivariate analyses are depicted in Supplementary Table S1, including multivariate hazard ratios, significance levels, and 95% confidence intervals.

### 3.4. KRT5 and KRT20 mRNA Expression in the MDACC Cohort

**Figure 3:**
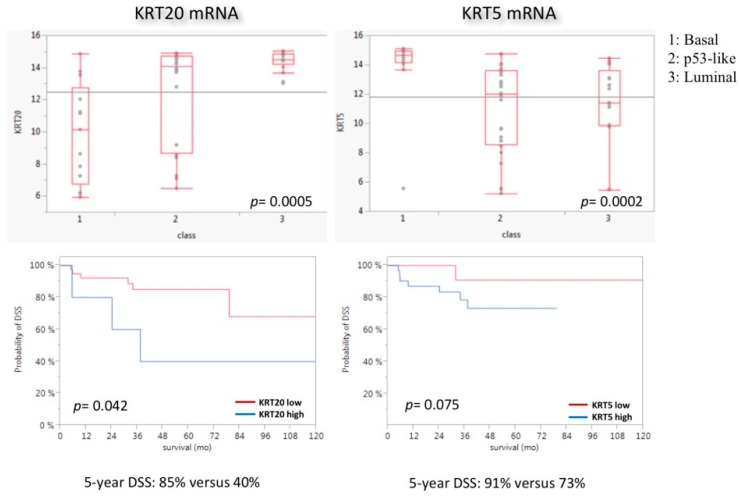
Association of *KRT5* and *KRT20* mRNA expression levels with MDACC molecular subtypes (MDACC cohort). Class 1 = basal, Class 2 = p53-like, Class 3 = luminal. As expected, luminal tumors were enriched with *KRT20* and basal tumors were enriched with *KRT5*. p53-like subtype shows a broad expression range of both genes. Basal tumors show a slightly higher proliferation rate. Kaplan–Meier regression analysis is unfavorable for highly *KRT20*-expressing tumors.

Data validation was performed using in silico MDACC data (GSE48276; GSE = gene set enrichment) [14]. The basal subtype was significantly enriched with *KRT5* (*p* = 0.0002), whereas the luminal subtype showed significant enrichment with *KRT20* (*p* = 0.0005) (Figure 3). High expression of *KRT20* mRNA was associated with significantly worse DSS, while *KRT5* mRNA expression had no prognostic impact (*p* = 0.042 for *KRT20* and *p* = 0.075 for *KRT5*; Figure 3). In addition, the Epi-Typer algorithm added no prognostic impact to the *KRT20*/*KRT5* cutoff (data not shown).

## 4. Discussion

Treatment options for UBC have evolved minimally over the last decades. Recent genome-wide mRNA expression analyses have revealed molecular subtypes with huge prognostic and predictive impact. Here, we show the possibility of stratifying MIBC into relevant subgroups by using two of the most prominent markers of the genome-wide approaches, the inversely related cytokeratins *KRT5* and *KRT20*, as surrogate markers for nonluminal and luminal differentiation. *KRT5* is a marker of stem or progenitor cells and can be found in basal-like carcinoma subtypes, often with squamous/sarcomatoid histological features, whereas *KRT20*, a marker of superficial umbrella cells, is enriched in luminal subtypes [8,9,12,13,14,15,17,20,26,27,28].

Most interestingly, the *KRT20* positive luminal subtype displayed worse RFS and DSS in MIBC, similar to previously published results in NMIBC [17,21]. This association between improved survival and keratin mRNA expression was also evident in the MDACC cohort (Figure 3) [14]. Furthermore, high expression of *KRT5* and low expression of *KRT20* were associated with a lower prevalence of lymphovascular invasion and lymphonodal metastasis, which is consistent with the favorable prognosis of *KRT5*-enriched MIBC in our cohort. At first glance, these results seem paradoxical, since the luminal subtype including *KRT20* was previously shown to be associated with a favorable prognosis [14], but are explainable due to characteristics of our cohort: (1) the *KRT20* high phenotype contains 23 cases with variant histology, of which are 13 micropapillary, 6 plasmacytoid, and 4 nested UBCs, which have been shown to be highly aggressive luminal variants with poor prognosis [29,30,31,32,33]; (2) many luminal tumors with and without variant histology are enriched with epithelial to mesenchymal transition (EMT) like gene expression pathways [18,29]. In a recent TCGA (=the cancer genome atlas) publication, Robertson et al. demonstrated that the luminal tumor family can be further subdivided into a luminal papillary cluster (no EMT-like pattern, favorable prognosis) and two luminal phenotypes with highly aggressive behavior (luminal, luminal infiltrated), which showed worse prognosis than basal differentiated tumors [18]. Luminal tumors with variant histology clustered into the aggressive luminal tumor families. Interestingly, Hedegaard et al. demonstrated that luminal NMIBC with poor PFS exhibited a strongly activated cancer-stem-cell-like and EMT signature and showed a huge parallel to the genomically unstable and infiltrated subtypes defined by the Lund group [13,17]. Additionally, in the past, several studies demonstrated the association between high *KRT20* expression and high tumor stage and grade [34]. High *KRT20* expression in lymph nodes after radical cystectomy is associated with a higher tumor stage, a higher rate of micrometastasis, and a worse outcome [35]. Moreover, high expression of *KRT20* in the bone marrow prior to radical cystectomy is associated with a worse outcome [36]. However, luminal tumors with favorable prognosis and *KRT20*/*KRT5* expression above the cutoff threshold are included in the favorable Epi-Typer class 1. This could mean that highly aggressive luminal tumors exhibit a strong *KRT20* polarized luminal-only phenotype with very low *KRT5* expression, while less aggressive luminal tumors show a mixed expression phenotype, reflecting differentiation that is still more closely related to the normal urothelial expression phenotype. Interestingly, we could prove that luminal variants exhibit significantly higher levels of *KRT20* and significantly lower levels of *KRT5* than conventional or basal variants (squamous/sarcomatoid). On the other hand, highly *KRT5*-expressing tumors are suggested to be of basal subtype and to respond better to neoadjuvant chemotherapy [5], which did not show worse survival than conventional luminal UBC in our cohort. However, the Epi-Typer algorithm is able to stratify class 1 basal and luminal tumors to identify patients who could benefit from neoadjuvant chemotherapy. Due to the lack of patients with neoadjuvant treatment in our cohort, the predictive potential of our RT-qPCR assay has to be investigated in an upcoming study.

As demonstrated previously in NMIBC, the assessment of cytokeratin (CK) 5 and CK20 protein expression by immunohistochemistry correlates well with *KRT5* and *KRT20* mRNA expression but lacks prognostic value [21], which is in line with previous breast cancer studies investigating *MKI67 (marker of proliferation Ki-67)*, *ER (estrogen receptor)*, *ERBB2 (Erb-B2 Receptor Tyrosine Kinase 2)*, and *PR (progesterone receptor)* mRNA and protein expression [37]. Therefore, RT-qPCR has been considered as a possible alternative for immunohistochemistry, as it is objective and not affected by interobserver variability [37,38,39,40]. Furthermore, simple gene expression assays in a ready-to-use format are quite simple to establish and to perform on small devices (e.g., Cepheid approaches) compared to immunohistochemistry on expansive autostainers. Tests on such platforms are very cheap and highly standardized, and need little hands-on time. Furthermore, no big laboratory inventory is needed to perform these ready-to-use assays, and therefore they are also suitable for small centers, private practices, or labs that do not have the opportunity to establish the extremely expensive infrastructure for next-generation sequencing or immunohistochemistry. On the other hand, there are several disadvantages with such tests: Due to the increased treatment individualization, they tend to oversimplify biological backgrounds. Furthermore, important predictive genetic alterations—e.g., microsatellite instability for response to checkpoint inhibition [41], recombinant DNA mismatch repair deficiency status for neoadjuvant chemotherapy [42], and others—are not assessable with such simple tests. Taken together, simple tests may play a big role in initial risk stratification to stratify which patients could benefit from further large-scale analysis after initial curative treatment.

Taken together, our data suggest that RT-qPCR-based molecular subtyping of UBC by *KRT5* and *KRT20* mRNA expression is a suitable method to predict RFS and DSS of MIBC patients (Epi-Typer) and could be used in small centers without access to huge immunohistochemistry facilities. Since our study is limited by its retrospective nature, small study cohort, and data from a single center, our results have to be further investigated in upcoming prospective trials with regard to specific treatment modalities.

## Figures and Tables

**Table 1:** Clinicopathological characteristics of the Mannheim cohort (overall and respective subgroups). KRT, keratin; L, lymphovascular invasion; V, blood vessel invasion; R, resection margin; WHO, World Health Organization; n.s., not significant; G, Grade; T, Tumor. * *p*-Value a: *KRT5_high_* vs. *KRT5_low_*; *p*-value b: *KRT20_high_* vs. *KRT20_low_*.

Characteristic	Total	*KRT5* High	*KRT5* Low	*KRT20* High	*KRT20* Low	*p*-Value
**Cohort size (n)**	122	89	33	48	74	
**Mean age (years)**	67.9	67.9	68.4	67.5	67.9	a: n.s. b: n.s.
**Gender (n)**						
*Male*	89 (73%)	66 (74%)	23 (70%)	34 (71%)	55 (74%)	a: n.s.
*Female*	33 (27%)	23 (26%)	10 (30%)	14 (29%)	19 (26%)	b: n.s.
**Adjuvant chemotherapy**	22 (18%)	14 (16%)	8 (24%)	9 (19%)	13 (17%)	n.s.
**Pathological characteristics**						
pTis (concomitant)	43 (35%)	34 (38%)	9 (27%)	19 (40%)	24 (32%)	a: n.s. b: n.s.
pT2	33 (27%)	28 (31%)	5 (15%)	14 (29%)	19 (26%)	a: 0.027
pT3	62 (51%)	44 (50%)	18 (55%)	25 (52%)	37 (50%)	b: n.s.
pT4	27 (22%)	17 (19%)	10 (30%)	9 (19%)	18 (24%)	
pN0	74 (60%)	60 (67%)	14 (42%)	20 (42%)	54 (73%)	a: 0.0005
pN1-2	48 (37%)	29 (33%)	19 (57%)	28 (58%)	20 (27%)	b: 0.002
L0	62 (51%)	52 (58%)	10 (30%)	17 (35%)	45 (61%)	a: 0.005
L1	60 (49%)	37 (52%)	23 (70%)	31 (65%)	29 (39%)	b: 0.006
V0	104 (85%)	81 (91%)	23 (70%)	42 (87%)	61 (84%)	a: 0.005
V1	18 (15%)	8 (9%)	10 (30%)	6 (13%)	12 (16%)	b: n.s.
R0	105 (86%)	77 (86%)	28 (85%)	42 (88%)	63 (85%)	a: n.s.
R1	17 (14%)	12 (14%)	5 (15%)	6 (12%)	11 (15%)	b: n.s.
**Grading**						
**WHO 1973**						
*G1*	0 (0%)	0 (0%)	0 (0%)	0 (0%)	0 (0%)	
*G2*	27 (22%)	21 (24%)	6 (18%)	11 (23%)	16 (22%)	a: n.s.
*G3*	95 (78%)	68 (76%)	27 (82%)	37 (77%)	58 (78%)	b: n.s.
**WHO 2004**						
*Low grade*	0 (0%)	0 (0%)	0 (0%)	0 (0%)	0 (0%)	a: n.s.
*High grade*	122 (100%)	89 (100%)	33(100%)	48 (100%)	74 (100%)	b: n.s.
**Characteristic**		**Epi-Typer Class 1**	**Epi-Typer Class 2**			***p*-Value**
**Cohort size (n)**		103	19			
**Mean age (years)**		67.9	71			n.s.
**Gender (n)**						
*Male*		75 (73%)	14 (74%)			n.s.
*Female*		28 (27%)	5 (26%)			
**Adjuvant chemotherapy**		17 (17%)	5 (26%)			n.s.
**Pathological T stage**						
pTis (concomitant)		37 (36%)	6 (31%)			n.s.
pT2						
pT3						
pT4						
**Pathological characteristics**						
pN0		69 (67%)	5 (26%)			0.0009
pN1-2		34 (33%)	14 (74%)			
L0		58 (56%)	4 (21%)			0.004
L1		45 (44%)	15 (79%)			
V0		90 (87%)	14 (74%)			n.s.
V1		13 (13%)	5 (26%)			
R0		89 (86%)	16 (84%)			n.s.
R1		14 (14%)	3 (16%)			
**Grading WHO 1973**						
*G1*		0 (0%)	0 (0%)			n.s.
*G2*		22 (21%)	5 (26%)			n.s.
*G3*		81 (79%)		14 (74%)		n.s.
**Grading WHO 2004**						
*Low grade*		0 (0%)		0 (0%)		n.s.
*High grade*		103 (100%)		19 (100%)		n.s.

Distribution of clinic-pathological determinants across the entire cohort and respective subgroups. *p*-value a: *KRT5_high_* vs. *KRT5_low_*; *p*-value b: *KRT20_high_* vs. *KRT20_low_*.

## Supplementary Materials

Supplementary materials can be found at http://www.mdpi.com/1422-0067/19/11/3396/s1.

## Abbreviations

UBC	urothelial bladder cancer
MIBC	muscle-invasive bladder cancer
CK	cytokeratin
KRT	keratin
MKI67	marker of proliferation Ki67
CALM2	calmodulin 2
FFPE	formalin-fixed paraffin-embedded
MDACC	MD Anderson Cancer Center
PFS	progression-free survival
RFS	recurrence-free survival
DSS	disease-specific survival
NMIBC	non-muscle-invasive bladder cancer
RT-qPCR	reverse-transcription quantitative polymerase chain reaction
