# Supplementary material for: mRNA-Expression of KRT5 and KRT20 Defines Distinct Prognostic Subgroups of Muscle-Invasive Urothelial Bladder Cancer Correlating with Histological Variants
Source: Int J Mol Sci. 2018 Oct 30;19(11):3396. doi: 10.3390/ijms19113396 (PMC6274690; doi:10.3390/ijms19113396)
Supplement: Supplementary file 1 [file ijms-19-03396-s001.zip › ijms-368875-supplementary.docx]

**Supplementary Table 1: Multivariate Hazard ratios of different *KRT5*, *KRT20* and Epi-Typer classes.**

**1) Multivariate Hazard Ratios: Disease specific survival – *KRT20* high vs. low (ad Figure 2A).**

| **Age** | | | | |  |
| --- | --- | --- | --- | --- | --- |
| **Term** | **Hazard Ratio** | **Lower 95%** | **Upper 95%** | **Reciprocal** |  |
| Age | 1,01 | 0,98 | 1,04 | 0,99 |  |
| **Age** | | | | |  |
| **Term** | **Hazard Ratio** | **Lower 95%** | **Upper 95%** | **Reciprocal** |  |
| Age | 1,43 | 0,38 | 5,65 | 0,70 |  |
| **WHO 1973 Grading** | | | | | |
| **Level1** | **/Level2** | **Hazard Ratio** | **Prob>Chisq** | **Lower 95%** | **Upper 95%** |
| G3 | G2 | 1,31 | 0,52 | 0,59 | 3,33 |
| G2 | G3 | 0,76 | 0,52 | 0,30 | 1,69 |
| **Gender** | | | | | |
| **Level1** | **/Level2** | **Hazard Ratio** | **Prob>Chisq** | **Lower 95%** | **Upper 95%** |
| male | female | 0,84 | 0,59 | 0,45 | 1,64 |
| female | male | 1,20 | 0,59 | 0,61 | 2,24 |
| **pT-Stage** | | | | | |
| **Level1** | **/Level2** | **Hazard Ratio** | **Prob>Chisq** | **Lower 95%** | **Upper 95%** |
| pT3 | pT2 | 2,08 | 0,09 | 0,90 | 5,24 |
| pT4 | pT2 | 3,10 | 0,03 | 1,09 | 9,36 |
| pT4 | pT3 | 1,49 | 0,29 | 0,70 | 3,10 |
| pT2 | pT3 | 0,48 | 0,09 | 0,19 | 1,11 |
| pT2 | pT4 | 0,32 | 0,03 | 0,11 | 0,91 |
| pT3 | pT4 | 0,67 | 0,29 | 0,32 | 1,42 |
| **pN-Stage** | | | | | |
| **Level1** | **/Level2** | **Hazard Ratio** | **Prob>Chisq** | **Lower 95%** | **Upper 95%** |
| pN0 | pN+ | 0,61 | 0,29 | 0,23 | 1,49 |
| pN+ | pN0 | 1,64 | 0,29 | 0,67 | 4,41 |
| **Lymphovascular Invasion** | | | | | |
| **Level1** | **/Level2** | **Hazard Ratio** | **Prob>Chisq** | **Lower 95%** | **Upper 95%** |
| 1 | 0 | 1,22 | 0,70 | 0,43 | 3,14 |
| 0 | 1 | 0,82 | 0,70 | 0,32 | 2,31 |
| **Blood Vessel Invasion** | | | | | |
| **Level1** | **/Level2** | **Hazard Ratio** | **Prob>Chisq** | **Lower 95%** | **Upper 95%** |
| 1 | 0 | 1,72 | 0,17 | 0,78 | 3,61 |
| 0 | 1 | 0,58 | 0,17 | 0,28 | 1,28 |
| **Resection Margin** | | | | | |
| **Level1** | **/Level2** | **Hazard Ratio** | **Prob>Chisq** | **Lower 95%** | **Upper 95%** |
| R1 | R0 | 1,20 | 0,65 | 0,52 | 2,54 |
| R0 | R1 | 0,83 | 0,65 | 0,39 | 1,94 |
| **Receipt of adjuvant platinum containing chemotherapy** | | | | | |
| **Level1** | **/Level2** | **Hazard Ratio** | **Prob>Chisq** | **Lower 95%** | **Upper 95%** |
| Yes | No | 0,91 | 0,81 | 0,43 | 1,90 |
| No | Yes | 1,09 | 0,81 | 0,53 | 2,35 |
| **Concomitant Carcinoma in situ** | | | | | |
| **Level1** | **/Level2** | **Hazard Ratio** | **Prob>Chisq** | **Lower 95%** | **Upper 95%** |
| No CIS | CIS | 0,56 | 0,06 | 0,30 | 1,04 |
| CIS | No CIS | 1,80 | 0,06 | 0,97 | 3,34 |
| **KRT20 high vs. Low** | | | | | |
| **Level1** | **/Level2** | **Hazard Ratio** | **Prob>Chisq** | **Lower 95%** | **Upper 95%** |
| KRT20>=37,9 | KRT20<37,9 | 2,24 | 0,01 | 1,19 | 4,25 |
| KRT20<37,9 | KRT20>=37,9 | 0,45 | 0,01 | 0,24 | 0,84 |

**2) Multivariate Hazard Ratios: Recurrence free survival – *KRT20* high vs. low (ad Figure 2A).**

| **Age** | | | | |  |
| --- | --- | --- | --- | --- | --- |
| **Term** | **Hazard Ratio** | **Lower 95%** | **Upper 95%** | **Reciprocal** |  |
| Age | 1,01 | 0,98 | 1,04 | 0,99 |  |
| **Age** | | | | |  |
| **Term** | **Hazard Ratio** | **Lower 95%** | **Upper 95%** | **Reciprocal** |  |
| Age | 1,57 | 0,44 | 5,69 | 0,64 |  |
| **WHO Grading 1973** | | | | | |
| **Level1** | **/Level2** | **Hazard Ratio** | **Prob>Chisq** | **Lower 95%** | **Upper 95%** |
| G3 | G2 | 1,35 | 0,42 | 0,66 | 3,06 |
| G2 | G3 | 0,74 | 0,42 | 0,33 | 1,51 |
| **Gender** | | | | | |
| **Level1** | **/Level2** | **Hazard Ratio** | **Prob>Chisq** | **Lower 95%** | **Upper 95%** |
| male | female | 0,58 | 0,09 | 0,32 | 1,08 |
| female | male | 1,72 | 0,09 | 0,92 | 3,14 |
| **pT-Stage** | | | | | |
| **Level1** | **/Level2** | **Hazard Ratio** | **Prob>Chisq** | **Lower 95%** | **Upper 95%** |
| pT3 | pT2 | 2,02 | 0,08 | 0,92 | 4,82 |
| pT4 | pT2 | 3,02 | 0,02 | 1,16 | 8,32 |
| pT4 | pT3 | 1,50 | 0,25 | 0,75 | 2,92 |
| pT2 | pT3 | 0,49 | 0,08 | 0,21 | 1,09 |
| pT2 | pT4 | 0,33 | 0,02 | 0,12 | 0,86 |
| pT3 | pT4 | 0,67 | 0,25 | 0,34 | 1,34 |
| **pN-Stage** | | | | | |
| **Level1** | **/Level2** | **Hazard Ratio** | **Prob>Chisq** | **Lower 95%** | **Upper 95%** |
| pN0 | pN+ | 0,63 | 0,29 | 0,25 | 1,45 |
| pN+ | pN0 | 1,58 | 0,29 | 0,69 | 3,93 |
| **Lymphovascular Invasion** | | | | | |
| **Level1** | **/Level2** | **Hazard Ratio** | **Prob>Chisq** | **Lower 95%** | **Upper 95%** |
| 1 | 0 | 1,22 | 0,67 | 0,47 | 2,91 |
| 0 | 1 | 0,82 | 0,67 | 0,34 | 2,11 |
| **Blood Vessel Invasion** | | | | | |
| **Level1** | **/Level2** | **Hazard Ratio** | **Prob>Chisq** | **Lower 95%** | **Upper 95%** |
| 1 | 0 | 2,27 | 0,03 | 1,11 | 4,50 |
| 0 | 1 | 0,44 | 0,03 | 0,22 | 0,90 |
| **Resection margin** | | | | | |
| **Level1** | **/Level2** | **Hazard Ratio** | **Prob>Chisq** | **Lower 95%** | **Upper 95%** |
| R1 | R0 | 0,97 | 0,93 | 0,44 | 1,96 |
| R0 | R1 | 1,03 | 0,93 | 0,51 | 2,29 |
| **Receipt of adjuvant platinum containing chemotherapy** | | | | | |
| **Level1** | **/Level2** | **Hazard Ratio** | **Prob>Chisq** | **Lower 95%** | **Upper 95%** |
| Yes | No | 1,28 | 0,49 | 0,63 | 2,53 |
| No | Yes | 0,78 | 0,49 | 0,39 | 1,59 |
| **Concomitant Carcinoma in situ** | | | | | |
| **Level1** | **/Level2** | **Hazard Ratio** | **Prob>Chisq** | **Lower 95%** | **Upper 95%** |
| No CIS | CIS | 0,64 | 0,14 | 0,35 | 1,17 |
| CIS | No CIS | 1,57 | 0,14 | 0,86 | 2,85 |
| **KRT20 high vs. Low** | | | | | |
| **Level1** | **/Level2** | **Hazard Ratio** | **Prob>Chisq** | **Lower 95%** | **Upper 95%** |
| KRT20>=37,9 | KRT20<37,9 | 2,33 | 0,01 | 1,29 | 4,23 |
| KRT20<37,9 | KRT20>=37,9 | 0,43 | 0,01 | 0,24 | 0,78 |

**3) Multivariate Hazard Ratios: Disease specific survival – *KRT5* high vs. low (ad Figure 2B).**

| **Age** | | | | |  |
| --- | --- | --- | --- | --- | --- |
| **Term** | **Hazard Ratio** | **Lower 95%** | **Upper 95%** | **Reciprocal** |  |
| Age | 1,00961 | 0,98 | 1,04 | 0,99 |  |
| **Age** | | | | |  |
| **Term** | **Hazard Ratio** | **Lower 95%** | **Upper 95%** | **Reciprocal** |  |
| Age | 1,584115 | 0,40 | 6,39 | 0,63 |  |
| **WHO 1973 Grading** | | | | | |
| **Level1** | **/Level2** | **Hazard Ratio** | **Prob>Chisq** | **Lower 95%** | **Upper 95%** |
| G3 | G2 | 1,14 | 0,77 | 0,51 | 2,91 |
| G2 | G3 | 0,88 | 0,77 | 0,34 | 1,98 |
| **Gender** | | | | | |
| **Level1** | **/Level2** | **Hazard Ratio** | **Prob>Chisq** | **Lower 95%** | **Upper 95%** |
| male | female | 0,92 | 0,80 | 0,50 | 1,80 |
| female | male | 1,08 | 0,80 | 0,56 | 2,01 |
| **pT-Stage** | | | | | |
| **Level1** | **/Level2** | **Hazard Ratio** | **Prob>Chisq** | **Lower 95%** | **Upper 95%** |
| pT3 | pT2 | 1,89 | 0,14 | 0,81 | 4,85 |
| pT4 | pT2 | 2,54 | 0,08 | 0,90 | 7,63 |
| pT4 | pT3 | 1,34 | 0,42 | 0,65 | 2,70 |
| pT2 | pT3 | 0,53 | 0,14 | 0,21 | 1,24 |
| pT2 | pT4 | 0,39 | 0,08 | 0,13 | 1,11 |
| pT3 | pT4 | 0,75 | 0,42 | 0,37 | 1,54 |
| **pN-Stage** | | | | | |
| **Level1** | **/Level2** | **Hazard Ratio** | **Prob>Chisq** | **Lower 95%** | **Upper 95%** |
| pN0 | pN+ | 0,44 | 0,06 | 0,17 | 1,02 |
| pN+ | pN0 | 2,27 | 0,06 | 0,98 | 5,76 |
| **Lymphovascular Invasion** | | | | | |
| **Level1** | **/Level2** | **Hazard Ratio** | **Prob>Chisq** | **Lower 95%** | **Upper 95%** |
| 1 | 0 | 1,15 | 0,79 | 0,41 | 3,00 |
| 0 | 1 | 0,87 | 0,79 | 0,33 | 2,45 |
| **Blood Vessel Invasion** | | | | | |
| **Level1** | **/Level2** | **Hazard Ratio** | **Prob>Chisq** | **Lower 95%** | **Upper 95%** |
| 1 | 0 | 1,30 | 0,49 | 0,60 | 2,66 |
| 0 | 1 | 0,77 | 0,49 | 0,38 | 1,66 |
| **Resection margin** | | | | | |
| **Level1** | **/Level2** | **Hazard Ratio** | **Prob>Chisq** | **Lower 95%** | **Upper 95%** |
| R1 | R0 | 1,21 | 0,63 | 0,53 | 2,54 |
| R0 | R1 | 0,82 | 0,63 | 0,39 | 1,90 |
| **Receipt of adjuvant platinum containing chemotherapy** | | | | | |
| **Level1** | **/Level2** | **Hazard Ratio** | **Prob>Chisq** | **Lower 95%** | **Upper 95%** |
| Yes | No | 0,85 | 0,68 | 0,39 | 1,80 |
| No | Yes | 1,17 | 0,68 | 0,55 | 2,57 |
| **KRT5 high vs. Low** | | | | | |
| **Level1** | **/Level2** | **Hazard Ratio** | **Prob>Chisq** | **Lower 95%** | **Upper 95%** |
| KRT5>=32,59 | KRT5<32,59 | 0,63 | 0,14 | 0,34 | 1,18 |
| KRT5<32,59 | KRT5>=32,59 | 1,59 | 0,14 | 0,85 | 2,92 |
| **Concomitant Carcinoma in situ** | | | | | |
| **Level1** | **/Level2** | **Hazard Ratio** | **Prob>Chisq** | **Lower 95%** | **Upper 95%** |
| No CIS | CIS | 0,48 | 0,02 | 0,25 | 0,91 |
| CIS | No CIS | 2,09 | 0,02 | 1,10 | 3,95 |

**4) Multivariate Hazard Ratios: Recurrence free survival – *KRT5* high vs. low (ad Figure 2B).**

| **Age** | | | | |  |
| --- | --- | --- | --- | --- | --- |
| **Term** | **Hazard Ratio** | **Lower 95%** | **Upper 95%** | **Reciprocal** |  |
| Age | 1,01 | 0,98 | 1,04 | 0,99 |  |
| **Age** | | | | |  |
| **Term** | **Hazard Ratio** | **Lower 95%** | **Upper 95%** | **Reciprocal** |  |
| Age | 1,54 | 0,42 | 5,82 | 0,65 |  |
| **WHO Grading 1973** | | | | | |
| **Level1** | **/Level2** | **Hazard Ratio** | **Prob>Chisq** | **Lower 95%** | **Upper 95%** |
| G3 | G2 | 1,21 | 0,62 | 0,59 | 2,74 |
| G2 | G3 | 0,83 | 0,62 | 0,36 | 1,70 |
| **Gender** | | | | | |
| **Level1** | **/Level2** | **Hazard Ratio** | **Prob>Chisq** | **Lower 95%** | **Upper 95%** |
| male | female | 0,65 | 0,16 | 0,36 | 1,20 |
| female | male | 1,54 | 0,16 | 0,83 | 2,76 |
| **pT-Stage** | | | | | |
| **Level1** | **/Level2** | **Hazard Ratio** | **Prob>Chisq** | **Lower 95%** | **Upper 95%** |
| pT3 | pT2 | 1,78 | 0,16 | 0,80 | 4,26 |
| pT4 | pT2 | 2,44 | 0,07 | 0,94 | 6,65 |
| pT4 | pT3 | 1,37 | 0,35 | 0,70 | 2,60 |
| pT2 | pT3 | 0,56 | 0,16 | 0,23 | 1,24 |
| pT2 | pT4 | 0,41 | 0,07 | 0,15 | 1,06 |
| pT3 | pT4 | 0,73 | 0,35 | 0,38 | 1,42 |
| **pN-Stage** | | | | | |
| **Level1** | **/Level2** | **Hazard Ratio** | **Prob>Chisq** | **Lower 95%** | **Upper 95%** |
| pN0 | pN+ | 0,46 | 0,05 | 0,19 | 1,00 |
| pN+ | pN0 | 2,18 | 0,05 | 1,00 | 5,16 |
| **Lymphovascular Invasion** | | | | | |
| **Level1** | **/Level2** | **Hazard Ratio** | **Prob>Chisq** | **Lower 95%** | **Upper 95%** |
| 1 | 0 | 1,17 | 0,74 | 0,46 | 2,81 |
| 0 | 1 | 0,86 | 0,74 | 0,36 | 2,20 |
| **Blood Vessel Invasion** | | | | | |
| **Level1** | **/Level2** | **Hazard Ratio** | **Prob>Chisq** | **Lower 95%** | **Upper 95%** |
| 1 | 0 | 1,84 | 0,09 | 0,91 | 3,58 |
| 0 | 1 | 0,54 | 0,09 | 0,28 | 1,10 |
| **Resection Margin** | | | | | |
| **Level1** | **/Level2** | **Hazard Ratio** | **Prob>Chisq** | **Lower 95%** | **Upper 95%** |
| R1 | R0 | 1,00 | 1,00 | 0,45 | 2,02 |
| R0 | R1 | 1,00 | 1,00 | 0,49 | 2,21 |
| **Receipt of adjuvant platinum containing chemotherapy** | | | | | |
| **Level1** | **/Level2** | **Hazard Ratio** | **Prob>Chisq** | **Lower 95%** | **Upper 95%** |
| Yes | No | 1,18 | 0,64 | 0,57 | 2,38 |
| No | Yes | 0,84 | 0,64 | 0,42 | 1,75 |
| **Concomitant Carcinoma in situ** | | | | | |
| **Level1** | **/Level2** | **Hazard Ratio** | **Prob>Chisq** | **Lower 95%** | **Upper 95%** |
| No CIS | CIS | 0,58 | 0,08 | 0,31 | 1,07 |
| CIS | No CIS | 1,74 | 0,08 | 0,94 | 3,19 |
| **KRT5 high vs. Low** | | | | | |
| **Level1** | **/Level2** | **Hazard Ratio** | **Prob>Chisq** | **Lower 95%** | **Upper 95%** |
| KRT5>=32,59 | KRT5<32,59 | 0,68 | 0,20 | 0,39 | 1,23 |
| KRT5<32,59 | KRT5>=32,59 | 1,47 | 0,20 | 0,81 | 2,58 |

**5) Multivariate Hazard Ratios: Disease specific survival – Epi-Typer classes (ad Figure 2C).**

| **Per unit change in regressor** | | | | |  |
| --- | --- | --- | --- | --- | --- |
| **Term** | **Hazard Ratio** | **Lower 95%** | **Upper 95%** | **Reciprocal** |  |
| Age | 1,01 | 0,98 | 1,04 | 0,99 |  |
| **Per change in regressor over entire range** | | | | |  |
| **Term** | **Hazard Ratio** | **Lower 95%** | **Upper 95%** | **Reciprocal** |  |
| Age | 1,34 | 0,34 | 5,41 | 0,75 |  |
| **WHO-Grading 1973** | | | | | |
| **Level1** | **/Level2** | **Hazard Ratio** | **Prob>Chisq** | **Lower 95%** | **Upper 95%** |
| G3 | G2 | 1,32 | 0,51 | 0,59 | 3,39 |
| G2 | G3 | 0,76 | 0,51 | 0,30 | 1,69 |
| **Concomitant Carcinoma in situ (urothelial)** | | | | | |
| **Level1** | **/Level2** | **Hazard Ratio** | **Prob>Chisq** | **Lower 95%** | **Upper 95%** |
| No CIS | CIS | 0,47 | 0,02 | 0,25 | 0,88 |
| CIS | No CIS | 2,14 | 0,02 | 1,13 | 4,04 |
| **Gender** | | | | | |
| **Level1** | **/Level2** | **Hazard Ratio** | **Prob>Chisq** | **Lower 95%** | **Upper 95%** |
| male | female | 0,87 | 0,67 | 0,47 | 1,70 |
| female | male | 1,15 | 0,67 | 0,59 | 2,14 |
| **pT-Stage** | | | | | |
| **Level1** | **/Level2** | **Hazard Ratio** | **Prob>Chisq** | **Lower 95%** | **Upper 95%** |
| pT3 | pT2 | 2,19 | 0,07 | 0,94 | 5,61 |
| pT4 | pT2 | 3,04 | 0,03 | 1,08 | 9,03 |
| pT4 | pT3 | 1,38 | 0,38 | 0,67 | 2,79 |
| pT2 | pT3 | 0,46 | 0,07 | 0,18 | 1,07 |
| pT2 | pT4 | 0,33 | 0,03 | 0,11 | 0,92 |
| pT3 | pT4 | 0,72 | 0,38 | 0,36 | 1,50 |
| **pN-Stage** | | | | | |
| **Level1** | **/Level2** | **Hazard Ratio** | **Prob>Chisq** | **Lower 95%** | **Upper 95%** |
| pN0 | pN+ | 0,53 | 0,16 | 0,20 | 1,27 |
| pN+ | pN0 | 1,88 | 0,16 | 0,79 | 4,90 |
| **Lymphovascular Invasion** | | | | | |
| **Level1** | **/Level2** | **Hazard Ratio** | **Prob>Chisq** | **Lower 95%** | **Upper 95%** |
| 1 | 0 | 1,15 | 0,78 | 0,41 | 2,99 |
| 0 | 1 | 0,87 | 0,78 | 0,33 | 2,45 |
| **Blood Vessel Invasion** | | | | | |
| **Level1** | **/Level2** | **Hazard Ratio** | **Prob>Chisq** | **Lower 95%** | **Upper 95%** |
| 1 | 0 | 1,47 | 0,32 | 0,67 | 3,03 |
| 0 | 1 | 0,68 | 0,32 | 0,33 | 1,48 |
| **Resection magin** | | | | | |
| **Level1** | **/Level2** | **Hazard Ratio** | **Prob>Chisq** | **Lower 95%** | **Upper 95%** |
| R1 | R0 | 1,10 | 0,82 | 0,48 | 2,30 |
| R0 | R1 | 0,91 | 0,82 | 0,43 | 2,10 |
| **Receipt of adjuvant platinum-containing chemotherapy** | | | | | |
| **Level1** | **/Level2** | **Hazard Ratio** | **Prob>Chisq** | **Lower 95%** | **Upper 95%** |
| Yes | No | 0,71 | 0,39 | 0,32 | 1,53 |
| No | Yes | 1,40 | 0,39 | 0,65 | 3,10 |
| **Epi-Typer Classes** | | | | | |
| **Level1** | **/Level2** | **Hazard Ratio** | **Prob>Chisq** | **Lower 95%** | **Upper 95%** |
| Class 2 | Class 1 | 3,20 | 0,00 | 1,53 | 6,47 |
| Class 1 | Class 2 | 0,31 | 0,00 | 0,15 | 0,65 |

**6) Multivariate Hazard Ratios: Recurrence free survival – Epi-Typer classes (ad Figure 2C).**

| **Age** | | | | |  |
| --- | --- | --- | --- | --- | --- |
| **Term** | **Hazard Ratio** | **Lower 95%** | **Upper 95%** | **Reciprocal** |  |
| Age | 1,01 | 0,98 | 1,04 | 0,99 |  |
| **Age** | | | | |  |
| **Term** | **Hazard Ratio** | **Lower 95%** | **Upper 95%** | **Reciprocal** |  |
| Age | 1,39 | 0,38 | 5,27 | 0,72 |  |
| **WHO Grading 1973** | | | | | |
| **Level1** | **/Level2** | **Hazard Ratio** | **Prob>Chisq** | **Lower 95%** | **Upper 95%** |
| G3 | G2 | 1,34 | 0,44 | 0,65 | 3,04 |
| G2 | G3 | 0,75 | 0,44 | 0,33 | 1,53 |
| **Gender** | | | | | |
| **Level1** | **/Level2** | **Hazard Ratio** | **Prob>Chisq** | **Lower 95%** | **Upper 95%** |
| male | female | 0,64 | 0,15 | 0,35 | 1,18 |
| female | male | 1,57 | 0,15 | 0,84 | 2,83 |
| **pT-Stage** | | | | | |
| **Level1** | **/Level2** | **Hazard Ratio** | **Prob>Chisq** | **Lower 95%** | **Upper 95%** |
| pT3 | pT2 | 1,92 | 0,11 | 0,87 | 4,60 |
| pT4 | pT2 | 2,58 | 0,05 | 1,01 | 6,98 |
| pT4 | pT3 | 1,34 | 0,38 | 0,69 | 2,54 |
| pT2 | pT3 | 0,52 | 0,11 | 0,22 | 1,15 |
| pT2 | pT4 | 0,39 | 0,05 | 0,14 | 0,99 |
| pT3 | pT4 | 0,74 | 0,38 | 0,39 | 1,45 |
| **pN-Stage** | | | | | |
| **Level1** | **/Level2** | **Hazard Ratio** | **Prob>Chisq** | **Lower 95%** | **Upper 95%** |
| pN0 | pN+ | 0,53 | 0,12 | 0,22 | 1,17 |
| pN+ | pN0 | 1,90 | 0,12 | 0,86 | 4,57 |
| **Lymphovascular Invasion** | | | | | |
| **Level1** | **/Level2** | **Hazard Ratio** | **Prob>Chisq** | **Lower 95%** | **Upper 95%** |
| 1 | 0 | 1,21 | 0,69 | 0,47 | 2,87 |
| 0 | 1 | 0,83 | 0,69 | 0,35 | 2,12 |
| **Blood Vessel Invasion** | | | | | |
| **Level1** | **/Level2** | **Hazard Ratio** | **Prob>Chisq** | **Lower 95%** | **Upper 95%** |
| 1 | 0 | 1,95 | 0,06 | 0,96 | 3,80 |
| 0 | 1 | 0,51 | 0,06 | 0,26 | 1,04 |
| **Resection Margin** | | | | | |
| **Level1** | **/Level2** | **Hazard Ratio** | **Prob>Chisq** | **Lower 95%** | **Upper 95%** |
| R1 | R0 | 0,98 | 0,95 | 0,44 | 1,97 |
| R0 | R1 | 1,03 | 0,95 | 0,51 | 2,26 |
| **Adjuvant Chemotherapy** | | | | | |
| **Level1** | **/Level2** | **Hazard Ratio** | **Prob>Chisq** | **Lower 95%** | **Upper 95%** |
| Yes | No | 1,05 | 0,89 | 0,50 | 2,16 |
| No | Yes | 0,95 | 0,89 | 0,46 | 2,01 |
| **Concomitant Carcinoma in situ** | | | | | |
| **Level1** | **/Level2** | **Hazard Ratio** | **Prob>Chisq** | **Lower 95%** | **Upper 95%** |
| No CIS | CIS | 0,58 | 0,08 | 0,32 | 1,07 |
| CIS | No CIS | 1,73 | 0,08 | 0,93 | 3,17 |
| **Epi-Typer Classes** | | | | | |
| **Level1** | **/Level2** | **Hazard Ratio** | **Prob>Chisq** | **Lower 95%** | **Upper 95%** |
| Class 2 | Class 1 | 2,10 | 0,04 | 1,02 | 4,18 |
| Class 1 | Class 2 | 0,48 | 0,04 | 0,24 | 0,98 |
